# Treatment of Rheumatism

**Published:** 1889-01

**Authors:** 


### TREATMENT OF RHEUMATISM.

A combination of salicylic acid and iron is recommended in the treatment of acute rheumatism, the formula for which was obtained in the the following way:

About a year ago a hospital nurse was pouring into a common receptacle some remnants of different medicines, when she noticed that a black precipitate formed by iron was turned into a transparent solution of a rich red hue, as soon as she poured the fluid contents of another bottle into it. Being a young woman of an inquiring turn of mind, she asked the house physician the cause of this phenomonon. The house staff, to help her in her desire for information, experimented with the drugs that she had been throwing out, and ascertained that her manipulation of chemicals had been this: she had first poured into the receptacle a salycilic acid. Into this she had poured a solution of iron, with the result of producing a black precipitate. To this she added some sodium phosphate, with the result of producing a clear red solution.

This at once gave a clue to the means of combining iron and salycilic acid without forming a precipitate. The facts were submitted to the apothecary of the hospital, and from them he produced the following formula; which has been in constant use nearly a year.

| | | |
|---|---|---|
| R. | Acidi salycili.......................... | gr. xx. |
| | Ferri pyrophosophatis.................... | gr. v. |
| | Sodii phosphatis....................... | gr. 1. |
| | Aquæ .................................... | oz. ss. |

